# Lymphocyte to monocyte ratio as a screening tool for influenza

**DOI:** 10.1371/currents.RRN1154

**Published:** 2010-03-29

**Authors:** George Merekoulias, Evangelos C Alexopoulos, Theodore Belezos, Eugenia Panagiotopoulou, Dr. med. Eleni Jelastopulu

**Affiliations:** ^*^University of Patras, Public health department; ^†^University of patras, Public health department; ^‡^ED Olympion Hospital; ^§^University of Patras and ^¶^Department of Public Health, School of Medicine, University of Patras

## Abstract

In fall 2009 the emergency department of a clinic in Greece with increased patient visits due to influenza-like illness observed a particular pattern in the complete blood count (CBC) of these patients. In 90% of all patients with probable influenza, lymphopenia and/or monocytosis were present. Relative lymphopenia with or without monocytosis appears to be a laboratory marker for H1N1 virus infection, a finding that could play a major role in early identifying and treating patients with new influenza A. A ratio of lymphocytes to monocytes below 2 is proposed as a screening tool for influenza infection instead of rapid tests.

## Introduction 

In the 20th century alone, there were three overwhelming pandemics, the Spanish flu in 1918, leading to 50 million deaths, Asian flu in 1957 (1 million deaths) and Hong Kong flu in 1968 (700.000 deaths), caused by H1N1, H2N2 and H3N2, respectively [1]. Avian flu (H5N1) was the first to mobilize the medical world and governments about the possibility of a new influenza pandemic [2], which is now a reality with the pandemic influenza A (H1N1) virus (swine flu), having already affected millions of people.

Recent and previous studies have revealed the clinical features of H1N1 virus infection [3] [4]. The mainly affected group is between 15 to 30 years old, the average incubation period is two days, and the majority of patients present with fever, cough, rhinorrhea, sore throat, myalgia and headache. Nausea, vomiting and diarrhoea are less frequent in adults compared to children [5].

For the uncomplicated cases, the disease is mild and no further invasive diagnostic testing is indicated [6], but preventive measures for the health care workers and guidelines for the patients are important, as the complications could be very serious, including acute respiratory distress syndrome and possible death even without comorbidities [7].

On the part of laboratory diagnostics the majority of patients reveal transient lymphopenia from the very first day [8]. Usually, a week after the symptoms subside, the virus cannot be traced [4].

The most sensitive but time-consuming evidence of infection with the new influenza A (H1N1) virus is provided by the reverse transcriptase polymerase chain reaction (RT-PCR) [9] as compared to the commercially available rapid tests for seasonal influenza, which show low sensitivity and specificity for H1N1 virus detection [10].

Therefore, confirming a diagnosis of H1N1 infection, even in hospitalized patients, can be proved difficult and protracted [6]. On the other hand starting antiviral treatment early  is recommended [11].

The aim of the present study was to examine, if there are more sensitive laboratory parameters, which could play a major role in identifying and treating those likely to have H1N1 virus infection.

## Material and methods

Data from the emergency department of a private clinic in Patras, Western Greece, were collected during the period from September to December 2009.Data included complete blood count (CBC), rapid test for influenza A/B (RIT), C reactive protein (CRP), demographic and clinical characteristics.

Study population included all patients visiting the emergency department during that period, with at least two of the following symptoms: fever, cough, rhinorrhea or sore throat and swollen lymph nodes. All patients whose diagnostic investigation revealed a disease other than a possible viral infection were excluded from the study.Thus, patients with lower respiratory tract infections and laboratory-confirmed elevated total white blood cells (WBC) and neutrophils, or patients with sore throats and positive strep test were excluded from the study as having in all probability bacterial infections. Furthermore, patients with detected IgM antibodies to Epstein-Barr virus (EBV) were excluded as well. 

Samples for the rapid test were taken by pharyngeal swab. SD BIOLINE one step influenza virus type A and B antigen tests were used. In the beginning all positive RITs were sent to specified laboratories for verification through RT-PCR, but later with the pandemic spread in November, the official guidelines required confirmation of hospitalized cases only. CBC was conducted by hematology analyzer CELL DYN 3700. Descriptive analysis was conducted using SPSS v 16.00.

## Results

The study population included 58 patients; the mean age was 28.6 years (range, 2 to 75), and 43% were male. More than 60% were aged 15 to 30 years, following the worldwide trend. The most common symptoms were cough (in 93% of the patients), fever (89%), rhinorrhea (54%) and swollen lymph nodes (20%).Incidence peaked at the end of November and early December with declining trends later on, in accordance to national data.

Table 1 presents the main laboratory parameters of the studied subjects. Normal white blood cell count (4100-10900/mm3) was observed in 90% of the patients. Lymphopenia (<1500/mm^3^ or <20 percent of total white blood cells) was present in 64% and monocytosis (>800/mm^3^ or >10 percent of total white blood cells) in 72% of the probable influenza cases. The majority of the patients with influenza-like illness (90%) revealed a decreased ratio of lymphocytes to monocytes (<2) with normal or low total white blood cells. 19 cases identified by RIT (33%) as positive, showed the latter laboratory characteristics (Table 1), and had a ratio of lymphocytes to monocytes in CBC near 2 or lower (only 2 cases ranged between 2,1-2,8). Thrombocytopenia was not observed, and CRP was slightly elevated.

There was only one complicated case,(lower tract infection), which needed to be hospitalised. Laboratory parameters revealed normal WBC and decreased ratio of lymphocytes to monocytes at the emergency department, while RT-PCR confirmed H1N1 virus infection.

**Table 1:** Laboratory parameters of all patients with influenza-like illness

**Table:** 

	**Patients with influenza-like illness (ILI)** **N=58**		
**Parameters**	**Total** **N=58**	**Positive Rapid Influenza Test (RIT)** **N=19**	** Lymphocytes/ monocytes ratio < 2** **Ν** **=39**
Leukocyte count Mean count (SD) <4000/mm^3^ >10900/mm^3^	7183 (2577) 5% 5%	6330 (1542) 0% 0%	6589 (2238) 5% 5%
Lymphocyte count Mean count (SD) <20% [lymphocyte/leukocyte] <1500/mm^3^	1709 (1298) 52% 64%	1191 (537) 63% 79%	1087 ( 478) 69% 87%
Monocyte count Mean count (SD) >10% [monocytes/leukocytes] >800/mm^3^	810 (325) 72% 43%	810 (278) 79% 47%	863 (306) 90% 51%
CRP (mg/dl ) Mean count (SD)	3 (2)	1,8 (1)	3 (2,3)
Rapid test for influenza A/B Influenza A Influenza B	32% 0%	100% 0%	48% 0%
Ratio < 2 ( Lymphocytes / Monocytes)	67%	85%	100%
Lymphopenia and/or Monocytosis	89%	100%	100%

## Discussion

According to recent studies lymphopenia is a common feature for seasonal influenza and new influenza A (H1N1) infection [4] [8]. It has been blamed for prolonged viral excretion and lower respiratory tract infection [12]. However, the presence of monocytosis observed in this study has not been evaluated up to now. In our study population, it is significantly related to positive RIT, especially when combined with relative or absolute lymphopenia, with a ratio of lymphocytes to monocytes below 2 (p<0.01).

During the current pandemic wave it is described that the majority of influenza infections in Greece are caused by the new H1N1 virus [13], and therefore this pattern on CBC could most likely be attributed to that virus.

Based on these observations we believe that monocytosis combined with lymphopenia, or a decreased ratio of lymphocytes to monocytes (<2), with normal or low total white blood cells, could trace more cases than the rapid influenza test , in patients with influenza like illness and be more useful if demand for virology testing exceeds capacity.

Taking into account the proposed ratio, 67% of all ILI cases in this study would be considered to be infected with H1N1 virus. The national data from the Hellenic center for disease control reported for the same period that 60% of all samples with influenza-like illness were testing positive for H1N1 virus by RT-PCR [13].

Given the fact that the hematology analyzer cannot discriminate between monocytes and large lymphocytes this study cannot suggest the presence of actual relative monocytosis, however the use of the relevant ratio in the emergency department is independent of that fact. A point score system for the probability of H1N1 infection, involving elements of CBC and clinical criteria has already been proposed for hospitalised patients [14]. At present, a decrease in the number of cases in Greece is observed, probably due to milder weather conditions. However, as lower temperatures in Greece are expected in March and April, a new pandemic wave could stress the health system again, considering the fact, that seasonal influenza shows usually a peak at the end of the winter months in Greece [13].

The latest update from WHO [15]  points that up to February 2010, worldwide more than 212 countries and overseas territories or communities have reported laboratory confirmed cases of pandemic influenza H1N1, including more than 15000 deaths, (4000 in Europe). In Europe, although pandemic influenza virus continues to circulate widely, particularly across central, southern, and eastern Europe, the overall intensity of pandemic influenza activity has declined substantially from peaks of activity seen earlier during the winter transmission period.There are more active areas of transmission like Northern Africa, South Asia, and East Asia [15].

However, even if a new pandemic wave does not occur, one should be still aware of the new influenza virus, because of its tendency to affect the lower respiratory tract [16]. Thus, a simple diagnostic tool as the proposed, can prove to be valuable in a low activity period, mainly in patients with  influenza-like symptoms.

Finally, a ratio of lymphocytes to monocytes below 2, is considered indicative of the ‘turn’ in the parameters of CBC. However, the optimal cut-off point should be determined based on the RT-PCR as gold-standard diagnostic test. 

We suggest this observation to be investigated in larger study populations including smaller age groups and performing RT-PCR and microscopic analysis of CBC in order to verify, if CBC and especially relative monocytosis could be applied as a time-saving and cost-effective screening test for H1N1 virus infection, leading to early antiviral treatment and hence to a decrease in the incidence of complicated cases. Such a tool would be very helpful in areas were laboratory confirmation is limited due to financial restrictions or excess demand.

## Competing interests

The authors declare having no competing interests. 

## Acknowledgements

The authors sincerely thank the medical personnel at the emergency department of the  Olympion Hospital, as well as the laboratory team for their assistance.

## Funding information

The authors declare no source of funding or other financial support.

## References

[ref-278273304] Hsieh YC, Wu TZ, Liu DP, Shao PL, Chang LY, Lu CY, et al. Influenza Pandemics: Past, Present and Future. Journal of the Formosan Medical Association 2006;105(1):1–6 10.1016/S0929-6646(09)60102-9 16440064

[ref-2528487683] Rajagopal S, Treanor J. Pandemic (Avian) Influenza. Seminars in Respiratory and Critical Care Medicine 2007; 28:159-170. 10.1055/s-2007-976488 17458770

[ref-2067255569] Kaji M, Watanabe A, Aizawa H. Differences in clinical features between influenza A H1N1, A H3N2, and B in adult patients. Respirology 2003; 8(2):231-3. 10.1046/j.1440-1843.2003.00457.x 12753540

[ref-1739006433] Cao B, Li XW, Mao Y, Wang J, Lu HZ, Chen YS, et al, for the National Influenza A Pandemic (H1N1) 2009 Clinical Investigation Group of China. Clinical Features of the Initial Cases of 2009 Pandemic Influenza A (H1N1) Virus Infection in China. New England Journal of Medicine 2009; 361:2507-17. 10.1056/NEJMoa0906612 20007555

[ref-4136871839] Jain R, Goldman R D. Novel Influenza A(H1N1) :Clinical Presentation, Diagnosis, and Management. Pediatric Emergency Care 2009; 25: 791-6. 10.1097/PEC.0b013e3181c3c8f8 19915434

[ref-871601418] Uyeki T. Diagnostic testing for 2009 pandemic influenza A (H1N1) virus infection in hospitalized patients. New England Journal of Medicine 2009;361:e114. 10.1056/NEJMopv0911052 19955517

[ref-346686940] The Australia and New Zealand Extracorporeal Membrane Oxygenation (ANZ ECMO) Influenza Investigators. Influenza A(H1N1) Acute Respiratory Distress Syndrome Extracorporeal Membrane Oxygenation for 2009.Journal Of the American Medical Association. 2009;302(17):1888-95. 10.1001/jama.2009.1535 19822628

[ref-3585927767] Cunha BA, Pherez F M, Schoch P. Diagnostic Importance of Relative Lymphopenia as a Marker of Swine Influenza (H1N1) in Adults. Clinical Infectious Diseases 2009; 49:1454–6. 10.1086/644496 19824851

[ref-3638083504] De Rosa FG, Montrucchio C, Di Perri G. Management of H1N1 influenza virus respiratory syndrome. Minerva Anestesiologica. 2009; 75(11):654-60. 19881461

[ref-3649321562] Faix DJ, Sherman SS, Waterman SH. Rapid-test sensitivity for novel swine origin influenza A (H1N1) virus in humans. New England Journal of Medicine 2009; 361(7):728-9. 10.1056/NEJMc0904264 19564634

[ref-250994421] Ruf BR, Szucs T. Reducing the burden of influenza-associated complications with antiviral therapy. Infection. 2009; 37(3):186-96. 10.1007/s15010-009-8241-1 19471854

[ref-1466456134] Gooskens J, Jonges M, Claas E.C.J, Meijer A, Kroes A.C.M. Prolonged influenza virus excretion during lymphocytopenia and frequent detection of drugresistant viruses. Oral presentations, ESCV 2009, Amsterdam / Journal of Clinical Virology 44 Suppl. 1 (2008) S5–S16. 10.1086/598684 19392620

[ref-3603145021] Hellenic center for disease control (KEELPNO) Accessed January 10, 2010. http://www.keelpno.gr/keelpno/2010/id994/gripi_ebdo_20100107.pdf

[ref-2032435731] Cunha BA, Syed U, Stroll S, Mickail N, Laguerre M. Winthrop-University Hospital Infectious Disease Division's swine influenza (H1N1) pneumonia diagnostic weighted point score system for hospitalized adults with influenza-like illnesses (ILIs) and negative rapid influenza diagnostic tests (RIDTs). Heart & Lung. 2009; 38(6):534-8. 10.1016/j.hrtlng.2009.09.005 PMC7119053 19944879

[ref-64086010] WHO Pandemic (H1N1) 2009 - update 87. Available at: http://www.who.int/csr/don/2010_02_12/en/index.html (Accessed February 15 2010)

[ref-2669711218] Yeh E, Luo RF, Dyner L, Hong DK, Banaei N, Baron EJ, Pinsky BA. Preferential lower respiratory tract infection in swine-origin 2009 A(H1N1) influenza. Clin Infect Dis. 2010 Feb 1;50(3):391-4. 20047483 10.1086/649875

